# Structurally differentiated *cis*-elements that interact with PU.1 are functionally distinguishable in acute promyelocytic leukemia

**DOI:** 10.1186/1756-8722-6-25

**Published:** 2013-04-02

**Authors:** Maoxiang Qian, Wen Jin, Xuehua Zhu, Xiaohong Jia, Xianwen Yang, Yanzhi Du, Kankan Wang, Ji Zhang

**Affiliations:** 1 Institute of Health Sciences, Shanghai Institutes for Biological Sciences, Chinese Academy of Sciences (CAS) & Shanghai Jiao Tong University School of Medicine (SJTU-SM), Shanghai, 200025, China; 2 State Key Laboratory of Medical Genomics and Shanghai Institute of Hematology, SJTU-SM, Shanghai, 200025, China; 3 Graduate School of the Chinese Academy of Sciences, Shanghai, 200031, China

**Keywords:** PU.1, PML/RARα, ChIP-seq, Acute promyelocytic leukemia, *cis*-element

## Abstract

**Background:**

Transcription factor PU.1, a member of the ETS family, is a master regulator of myeloid differentiation whose functional disruption is often associated with acute myeloid leukemia (AML). Although much has been learned about PU.1 over the past decades, relatively little is known about *cis*-elements that interact with this factor under physiological or pathological conditions, especially in the whole-genome scale. We aimed to define the cistrome of PU.1 in acute promyelocytic leukemia (APL) cells and characterize the *cis*-elements bound by PU.1.

**Methods:**

Chromatin immunoprecipitation with specific antibody coupled with deep sequencing (ChIP-seq) was used to investigate the *in vivo* PU.1 binding sites at the whole-genome scale in APL-derived NB4 cells. The ChIP-quantitative (q)-PCR and luciferase reporter assays were used to validate the binding events and *trans*-activity, respectively. Various computational analyses, including motif mining, evolutionary conservation analysis and functional enrichment analysis, were performed to characterize the *cis*-elements that interacted with PU.1.

**Results:**

A total of 26,907 significantly enriched binding regions of PU.1 were identified under the false discovery rate 0.1% in NB4 cells. PU.1 bound to various types of genomic regions and acted as a promoter-enhancer dual binding transcription factor. Based on the sequence length and composition, two types of representative motifs were identified in PU.1 binding sites: a long and a short motif. The long motif, characterized by high sequence specificity and binding affinity, predominantly resided in the promoter-distal regions. In contrast, the short one, with strong evolutionary constraint, represented the primary PU.1 *cis*-elements in the promoter-proximal regions. Interestingly, the short one showed more preference to be correlated with the binding of other factors, especially PML/RARα. Moreover, genes targeted by both PU.1 and PML/RARα were significantly involved in categories associated with oncogenesis, hematopoiesis and the pathogenesis of acute myeloid leukemia.

**Conclusions:**

Our results demonstrate that structurally differentiated *cis*-elements that interact with PU.1 are functionally distinguishable in APL, suggesting that the sequence diversity of *cis*-elements might be a critical mechanism by which cells interpret the genome, and contribute to distinct physiological and/or pathological function.

## Background

Biological processes are orchestrated by precise temporal and spatial regulation of gene expression, requiring proper interactions between *cis*-regulatory elements and *trans*-acting factors (TFs). Disruption of such interactions often causes disease. Over the past decades, much has been learned about actions of TFs under various physiological or pathological conditions, whereas relatively little is known about the *cis*-elements that control TF-specific gene expression [1]. Recent advances in genomic technologies, including chromatin immunoprecipitation coupled with deep sequencing (ChIP-seq), have allowed the genome-wide recognition of *in vivo cis-trans* interacting sites, thus facilitating the survey of structural and functional features of thousands of *cis*-elements simultaneously, and providing the opportunity to understand the mechanism of gene regulation in a more comprehensive manner.

In hematopoiesis, a number of master TFs which play major instructive roles for hematopoietic development or malignant transformation have been identified and intensively studied. PU.1, one of these master TFs, is exclusively expressed in hematopoietic cells and has been identified as a crucial transcription factor in normal hematopoiesis and in generation of myeloid leukemia through disruption of its function [2]. In acute promyelocytic leukemia (APL), a subtype of AML with the typical promyelocytic leukemia-retinoic acid receptor α (PML/RARα) fusion protein in the disease cells, PU.1 is expressed at reduced levels and increased expression mediated by gene transfer of PU.1 is sufficient to induce neutrophil differentiation, similar to the effect of all*-trans* retinoid acid (ATRA) [3,4]. With transgenic mouse models, the penetrance rate of APL development is significantly increased in offspring when PML/RARα mice are crossed with PU.1^+/-^ mice [5]. These observations collectively suggested the presence of crosstalk between PU.1 and PML/RARα in APL. Using ChIP combined with whole-genome promoter arrays, we previously investigated the early molecular effects of PML/RARα in hematopoietic progenitor cells and demonstrated that PML/RARα disrupts the PU.1 regulated genes and thus results in a blockage of the downstream PU.1 signaling [6]. However, a question regarding whether *cis*-elements that interact with PU.1 contribute to the selective binding of PML/RARα in APL remains unknown.

It is interesting to note that myeloid specific genes such as granulocyte colony-stimulating factor (G-CSF) receptor [7], granulocyte-macrophage (GM)-CSF receptor α [8] and macrophage (M)-CSF receptor [9] have PU.1 binding sites in their promoter regions. However, as these genes are investigated at the single-gene level, it is challenging to gain a comprehensive understanding of the *cis*-elements that are essential for the regulation of myeloid-specific genes. For instance, PU.1 was first reported to bind to purine-rich 5^′^-GGAA/T-3^′^ sequences, similar to other members of the Ets family [10,11]. However, later studies showed that the DNA binding specificity of PU.1 was quite different from that of the other members, in that some sites required a string of adenosine residues at the 5^′^ of the GA core [8,9,12,13]. Additional data indicated that some of the PU.1 binding sites were biologically significant but lack of the adenosine string at the 5^′^ of GA core [14]. Also, nucleotides (i.e., G and T) flanking the 3^′^ end of the GAGGAA sequence appear to be important for PU.1 binding and its transactivation activity [15]. Given the reported variability of PU.1 binding sites, and our interest in this transcription factor in disease, we performed ChIP-seq experiments in the APL-derived NB4 cells using PU.1-specific antibody to identify the *in vivo* PU.1 binding sites at the whole-genome scale. This revealed a number of interesting features which are potentially important for regulating myeloid differentiation and leukemogenesis.

## Results

### Identification and validation of *in vivo* binding regions of PU.1

**Figure 1:**
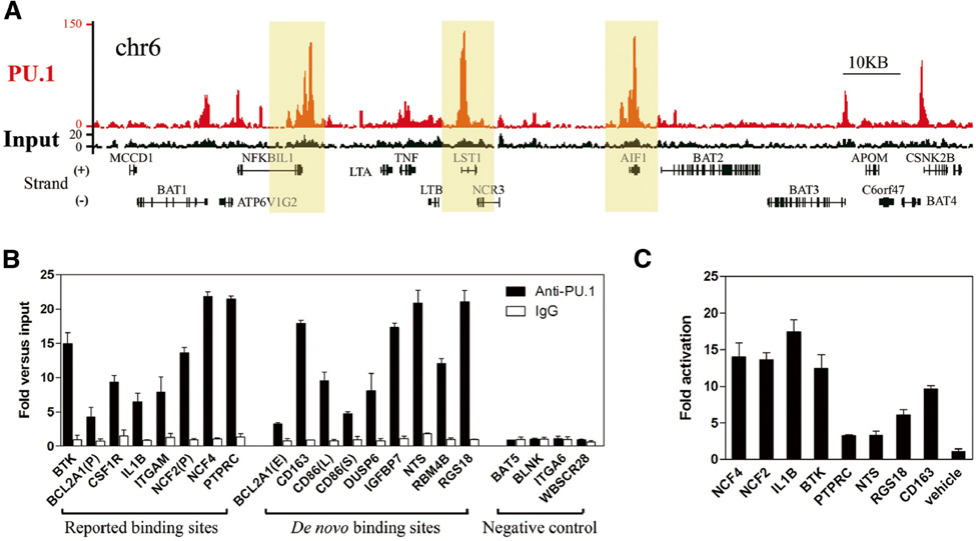
**Identification and validation of PU.1 binding regions.** (**A**) The visual representation of PU.1 targets on Chromosome 6 (chr6) partial regions identified through ChIP-seq analysis. The red and black tracks represent the ChIP-seq tag density of the sample (PU.1) and control (Input), respectively. (**B**) Validation of the PU.1 binding regions by ChIP-qPCR assays. Corresponding RefSeq genes for individual binding regions are marked underneath. Special codes “P” “E” “S” “L” inside the brackets, representing promoter, enhancer, short transcript and long transcript, were used to distinguish the regions corresponding to the same gene. (**C**) Luciferase reporter assays on representatives of PU.1 target genes (NCF4, NCF2, IL1B, BTK, PTPRC, NTS, RGS18, and CD1163) reporter plasmids and expression plasmids were co-transfected into 293T cells.

Chromatin immunoprecipitation (ChIP) with PU.1-specific antibody followed by deep sequencing was performed for the APL-derived NB4 cells. As shown in Table 1, a total of 15.6 million 35-bp sequence reads were generated, of which 11.8 million (76%) were aligned uniquely and non-redundantly to the human genome (HG18). Based on the false discovery rate (FDR) of 0.1%, a total of 26,907 significantly enriched ChIP regions with a median length of 429 bp were identified (Table 1, Additional file 1: Figure S1 and Additional file 2: Table S1) through MACS [16]. Figure 1A illustrates a representative chromatin region (6p21.33), showing enriched peaks of PU.1 binding. As shown in Figure 1A and Additional file 3: Figure S2, sharp enrichment peaks were found in proximal or/and distal regions of promoters for previously reported PU.1 target genes (e.g., *BTK*[17], *CSF1R*[9], *CSF2RB*[18], *ITGAM*[12], *NCF2*[19] and *NCF4*[20]), and novel target genes of PU.1, such as *NFKBIL1*, *LST1* and *AIF1*. As a validation, ChIP-quantitative(q)-PCR was then conducted on the binding regions of 8 previously known targets, 9 randomly selected *de novo* targets and 4 negative controls (Figure 1B), showing results consistent with those of ChIP-seq in this setting. Since PU.1 primarily is considered as a transactivator in myeloid differentiation [21], we then selected eight enriched regions on a random basis and applied them to luciferase assays in 293T cells (Figure 1C). Clearly, PU.1 transactivated these regions, adding further evidence that the ChIP regions identified in this setting represent *bona fide* functional binding sites of PU.1 in APL cells.

**Table 1:** ChIP-seq reads and peaks threshold at FDR=0.001

	**Parameter**	**NB4 PU.1**
Reads	Total sequenced (millions)	15.6
	Total, mapped (millions)	14.8
	Total, uniquely mapped and Non-redundant (millions)	11.8
	In peaks (millions)	1.44 (12.1%)
	Peak coverage (Mb)	13.1 (0.5%)
	Median width (bp)	429
	Enrichment	25.01
Peaks	Number of peaks	26,907
	Minimum fold enrichment	3.68
	Average fold enrichment	32.92
	Median fold enrichment	26.45

Notes: Peak coverage percentages assume a 2.7 Gb genome.

### Characterizing PU.1 as a promoter-enhancer dual binding TF

**Figure 2:**
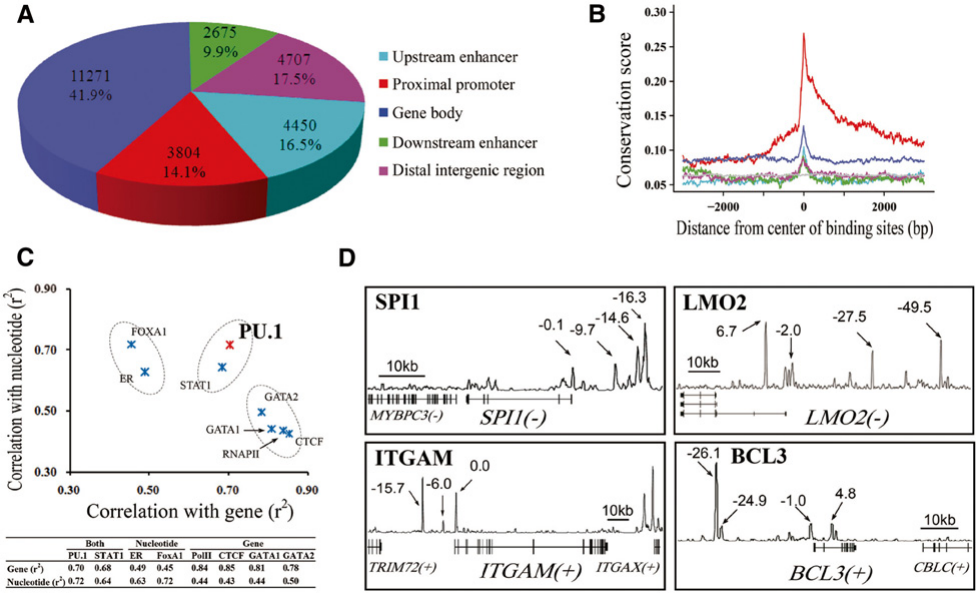
**Characterization of identified PU.1 binding regions.** (**A**) Pie diagram showing distribution of PU.1 binding regions located in the proximal promoter (within 2 kb 5^′^ and 1kb 3^′^ to the TSS), gene body (+1kb of TSS to transcription end site, TES), upstream enhancer (between at most -50 kb and -2 kb from TSS), downstream enhancer (from TES to at most 50 kb downstream), or distal intergenic regions (more than 50 kb to TSS and TES). (**B**) Evolutionary conservation analysis of each genomic type of PU.1 binding regions used multiple alignments of 27 vertebrate genomes with human. All the regions are aligned at peak summits in a 5^′^-to-3^′^ manner. (**C**) Correlation of the binding sites of PU.1, STAT1, ER, FOXA1, RNAPII, CTCF, GATA1 and GATA2 with each chromosome, ranked according to total gene number and total nucleotide number. (**D**) Representative PU.1 target genes identified through ChIP-seq analysis. (Arrows) ChIP-seq peak locations relative to the transcription start site of the respective PU.1 target gene (kb).

In an attempt to identify potential features associated with PU.1 binding sites, we first compared binding locations to annotated genes based on the UCSC Genome Browser RefSeq Database [22], and found that 14.1% (3,804/26,907) of the binding sites mapped to the promoter regions, 41.9% (11,271/26,907) to the intragenic regions (gene body) and 44.0% (11,832/26,907) to the intergenic regions (including 16.5% upstream enhancers, 9.9% downstream enhancers and 17.5% distal intergenic regions) (Figure 2A), as classified by the recommended criteria (see Materials and methods). These results indicate that the binding spectrum of PU.1 is highly complex and versatile in APL cells. Next, we conducted sequence evolutionary conservation analysis across 27 vertebrate genomes on each of the mapped sections. As shown in Figure 2B, the summit of PU.1 enriched binding regions revealed higher evolutionary conservation than the flanking regions, implicating the biological relevance of PU.1 binding. Moreover, the highest conservation scores were obtained with the promoter-proximal binding sites, supporting the notion that *cis*-elements in promoter regions are evolutionally conserved [23]. In contrast, the PU.1 promoter-distal binding sites (including those in the intragenic and intergenic regions) revealed relatively low conservation scores, consistent with the idea that *cis*-elements in enhancers or other distal regulatory elements are relatively dynamic among species [24].

Next, we performed a correlation analysis between the PU.1 binding sites versus total gene number and nucleotide number on each chromosome, respectively. As a control, we conducted the same analysis with the binding sites of a classic promoter-binding factor RNA polymerase II (RNAPII), a typical enhancer-binding protein estrogen receptor (ER) [25] and some other factors including CTCF [26], STAT1 [27], FOXA1 [28], GATA1 [29] and GATA2 [30] (Figure 2C and Additional file 4: Table S2), whose genome-wide binding sites have been documented previously. Interestingly, the PU.1 binding sites were correlated with both the chromosomal gene number (*r*^*2*^=0.70) and nucleotide number (*r*^*2*^=0.72) (Figure 2C), which appeared to be distinguishable from most of the other tested factors except for STAT1, a known promoter-enhancer dual binding TF [31,32]. The factors like RNAPII, CTCF, GATA1 and GATA2, were obviously correlated with the number of genes, whereas those like ER and FOXA1, known as two specific enhancer-binding proteins, were correlated with the number of nucleotides. The above observations suggest that PU.1 may act as a versatile factor able to interact with *cis*-elements not only in promoter regions but also in enhancer regions.

In addition, we investigated the PU.1 binding locations and numbers on their corresponding RefSeq genes (9,556), revealing that 33.3% (3,184/9,556) of the RefSeq genes harbored the binding sites on their promoter-proximal regions whereas 66.7% (6,372/9,556) contained the binding sites on the promoter-distal regions. These observations, together with data shown in Figure 2A, suggest that promoter-distal binding of PU.1 may play at least as equally important roles as the promoter-proximal binding in transcriptional regulation. Interestingly, more than half of the genes (1,598/3,184) with promoter-proximal binding of PU.1 appeared to contain additional binding sites in their promoter-distal regions, suggesting that PU.1 regulatory mechanisms can be far more complex than previously recognized, by involving multiple *trans*-*cis* interaction sites. This would allow for precise control of gene expression that is essential for myeloid differentiation. Indeed, auto-regulation of the PU.1-encoding gene *SPI1* appears to require PU.1 binding at both the promoter [21] and enhancer (i.e., 17 kb upstream) [33]. In this study, we found in addition to the sites reported previously two additional sites were identified 9.6 kb and 14.6 kb upstream of the gene (Figure 2D). An additional example is the integrin alpha M chain (CD11b) gene, *ITGAM*. This gene is known to be important for the adherence of neutrophils and monocytes during differentiation. It contains one PU.1 binding site at the promoter as previously reported [12]. In our study we have identified another site 16 kb upstream of the gene, representing a potential enhancer region (Figure 2D). Such structures may typically represent PU.1-involved *trans*-*cis* interactions required for myeloid differentiation. In consistent with this notion, numerous other myeloid differentiation-required genes are also multi-targeted by PU.1, such as LMO2, BCL3, IL1B and IL12B (Figure 2D and Additional file 5: Figure S3).

### Distinct features of the short and long motifs in the PU.1-bound regions

**Figure 3:**
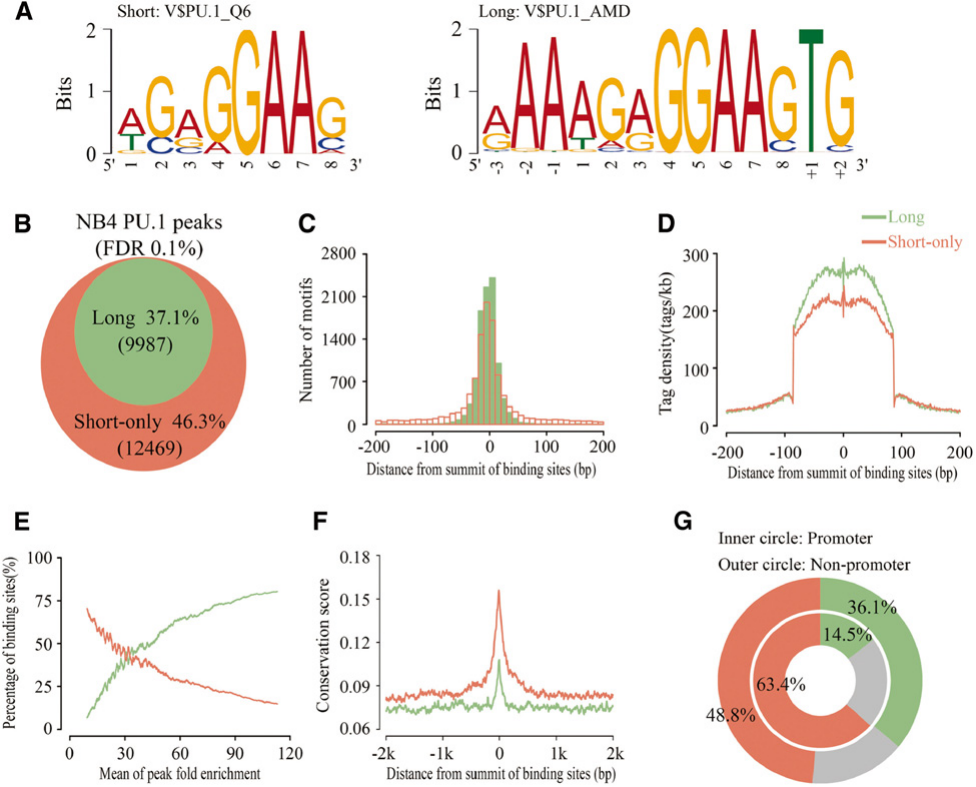
**Identification and characterization of enriched motifs within PU.1 binding regions.** (**A**) Sequence logos were shown for significant PU.1 motifs. The logo on the left was generated with V$PU.1_Q6 PSFM from TRANSFAC database, where the logo on the right was obtained by *de novo* motif finding using AMD. (**B**) Percentages of the PU.1 binding regions with the long motif (green) or the short motif only (orange). These two colors were consistently used to indicate the two specific sub regions in the following figure parts, respectively. (**C**) Histogram representing the distribution of the long PU.1 motif and only the short one within the PU.1 binding regions relative to the summit of the binding regions (represented as 0). (**D**) Comparison of tag density between the PU.1 binding regions with the long motif and only the short one. (**E**) Comparison of percentage of PU.1 binding sites between the long motif-containing and only the short motif-containing along the mean of peak fold enrichment. (**F**) Conservation analysis of PU.1 binding regions with the long motif or the short one used multiple alignments of 27 vertebrate genomes with Human. All of the regions are aligned at peak summits in a 5^′^-to-3^′^ manner. (**G**) Proportions of the long and only short motif-containing PU.1 binding sites from the promoter (inner circle) or non-promoter (outer circle) regions showed with annular charts. The proportion of neither the long nor short motif-containing PU.1 binding regions was indicated with the gray color.

To identify *cis*-elements that interact with PU.1 in the enriched binding regions, we conducted motif discovery analysis using several discovery tools, including (1) the *de novo* motif discovery methods AMD [34] and MEME [35], and (2) the prior-compiled PSFM-based motifs detection method termed MotifScan (see Materials and methods). Based on sequences corresponding to the top 500 most-enriched binding regions, two types of motifs appeared to be repeatedly observed in a significant manner (Figure 3A). One is contained in the database of TRANFAC and known as the canonical PU.1 consensus sequence of 5^′^-AG(A/G)GGAAG-3^′^ (left panel) and the other is found by *de novo* scanning*,* with a motif sequence of 5^′^-(A/G)AAAG(A/G)GGAAGTG-3^′^ (right panel and Additional file 6: Figure S4). This *de novo* motif covers the canonical one but identifies additional preferences including adenine at the -3 to -1 position, and thymine and guanine at the +1 and +2 positions. For convenience, we have named the motif including 5^′^ adenines as “long motif” and the canonical one as “short motif”.

Next, we scanned the total binding regions of PU.1 using the above long and short motifs by MotifScan. As shown in Figure 3B, 37.1% of the binding regions contained one or more long motifs, and 46.3% of the binding regions contained only the short motif. The remaining 16.6% revealed neither long nor short motifs, which were likely due to undetected PU.1-binding motifs present in these regions, or due to the possibility that for these sites PU.1 does not directly bind to chromatin, but rather forms a complex through protein-protein interactions. Positional distribution analysis revealed that both motifs, especially the long one, appeared to reside near the center of the binding regions (Figure 3C). We then evaluated the binding affinities of the long and short motifs by comparing their enrichment levels. As shown in Figure 3D, the long motif exhibited a much higher mean tag density than the short one, particularly with respect to the tag density in the regions (-100 to +100) flanking the summit of peaks. Consistently, we found that the higher the enrichment levels, the more (less) the percentages of the long (short) motifs (Figure 3E). These observations suggest that the long motif exhibits higher binding affinity to PU.1 than the short one. Interestingly, sequence evolutionary conservation analysis showed that the binding sites with the short motif appeared to be much more conserved than the long motif-containing sites (Figure 3F). These results together suggest that the motif preference may correlate with the motif location in the genome, implicating that functional roles played by the two types of motifs can be different in general. Then, we examined the proportional distributions of the short or long motifs in promoter regions vs. non-promoter regions, respectively. As shown in Figure 3G, the percentage of short or long motifs (48.8% vs. 36.1%, outer circle) in non-promoter regions was equivalent to that for the total of PU.1 binding sites (46.3% vs. 37.1%, Figure 3B), whereas, that in the promoter regions with 63.3% short motifs and 14.5% long motifs (inner circle) was significantly different from that.

In sum, *cis*-elements that interact with PU.1 can be classified into short and long motif classes based on their sequence patterns. In promoter regions, PU.1-*cis* elements are predominantly represented by those in the class of short motif, which are highly conserved across species but with lower binding affinity to PU.1. In contrast, *cis*-elements in the class of long motif are relatively depleted from promoter regions whereas primarily present in non-promoter regions, representing PU.1 binding sites of high affinity but evolutionally less conserved.

### Short motif-containing binding regions of PU.1 preferentially targeted by other factors including PML/RARα

**Figure 4:**
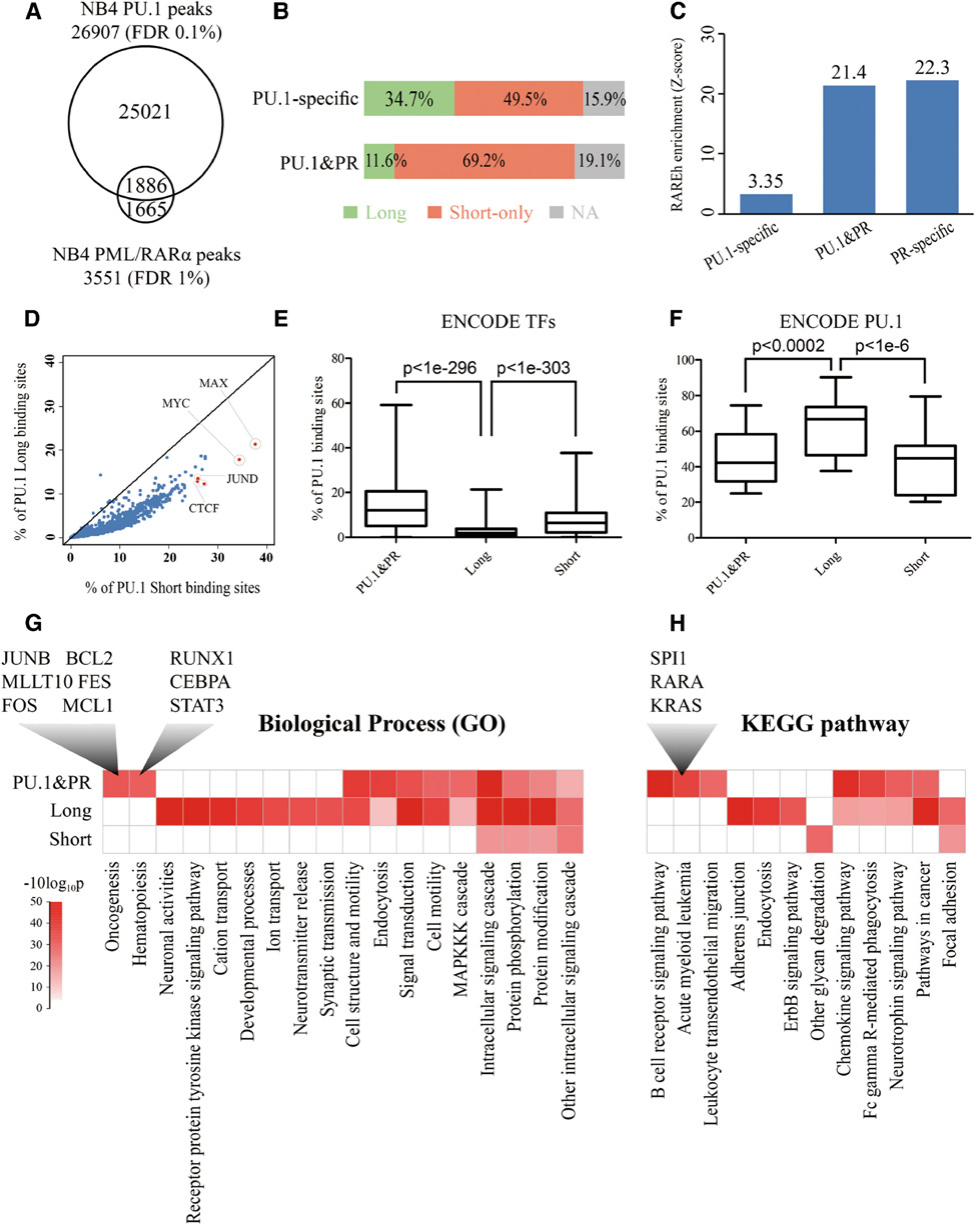
**PU.1 binding sites with short motif selectively targeted by other factors including PML/RARα.** (**A**) Venn diagram displaying the overlap of PU.1 binding regions and PML/RARα binding regions in NB4 cells. (**B**) Schematic illustration of the PU.1 motif comparison between PU.1 binding regions and PML/RARα binding regions. PU.1-specific is the subpopulation of PU.1 binding regions that do not overlap with PML/RARα binding regions. PU.1&PR is the subpopulation of PU.1 binding regions that overlap with PML/RARα binding regions. The color bars indicate the percentages of the regions with the long, only short PU.1 motif and neither, with green, orange and gray, respectively. (**C**) Bar plot of Motif RARE half (RAREh) enrichment in three subpopulations. PU.1-specific and PU.1&PR mean the same as above. PR-specific is the subpopulation of PML/RARα binding regions that do not overlap with PU.1 binding regions. (**D**) Scatter plot showing the covered percentages of PU.1-specific binding sites with the long motif versus that with the short motif by ENCODE ChIP-seq data sets of transcription factors except PU.1. (**E** and **F**) Box plot of the covered percentages in three PU.1 binding sets, including PU.1&PR, Long and Short, by ENCODE ChIP-seq data sets of PU.1 and other factors, respectively. The “Long” represents the PU.1-specific binding set with the long motif, while the “Short” represents those only with the short motif. The differences between the two binding sets were assessed using the paired t-test. The p-values are shown in the panels. (**G** and **H**) Heat map of functional enrichment with the items of PANTHER gene ontology in biological process and KEGG pathways. The enrichment level (-10*log10 (p-value)) was indicated with red color. The “PU.1&PR”, “Long” and “Short” represent the corresponding gene sets to the sets of binding sites defined above.

We previously demonstrated that PML/RARα selectively targets PU.1 binding regions that harbored both PU.1 binding sites and RARE (retinoic acid response element) half (RAREh) sites [6]. In the present study, we found that *cis*-elements that interact with PU.1 could be distinguished by the sequence length and composition, evolutionary conservation, genomic distribution and binding affinity. A remaining question would be whether these motifs were functionally differentiated, e.g., in the genesis of APL. In an attempt to answer this question, we examined PU.1 binding sites in the genomic regions targeted by PML/RARα. First, ChIP-seq was performed using specific antibodies against PML and RARα respectively, and a total of 3,551 highly significant (FDR < 0.01) PML/RARα binding sites were identified (Additional file 7: Table S3). When the binding sites of PML/RARα and those of PU.1 were compared, over 53% (1,886/3,551) of the PML/RARα were also targeted by PU.1 (Figure 4A). When short and long elements of PU.1 motifs were respectively compared in the PU.1-specific binding sites, and PU.1 and PML/RARα overlapping binding sites (PU.1&PR), we observed dramatic differences (Figure 4B). For the PU.1&PR set, almost 70% of the sites were represented by the short PU.1 motif while less than 12% were typified by the long motif. In contrast, the proportion in the PU.1-specific set was 49.5% long and 34.7% short. These results indicated that the short motif elements are strongly correlated with the selective targeting of PU.1 binding sites by PML/RARα whereas the long motif elements are largely depleted from such a targeting. Although much remains to be elucidated, our results, at least, suggest that binding of PU.1 to short motif elements may offer this factor a selective preference to recruit PML/RARα. Previously, we have shown that PU.1 is able to direct the binding of PML/RARα to nearby RARE half (RAREh) sites at the level of promoters [6]. We thus extended this analysis to a whole genome scale and analyzed the enrichment of RAREh sites in the three subpopulations (PU.1-specific, PU.1&PR and PR-specific). As illustrated in Figure 4C, RAREh sites were significantly enriched in the PR-specific (Z-score = 22.3) and PU.1&PR (Z-score = 21.4) sets, whereas the RAREh sites were relatively depleted from the PU.1-specific set (Z-score = 3.35). This result further indicates that the selective binding of PML/RARα to PU.1 binding sites requires both RAREh and short consensus elements of PU.1.

Next, we wanted to know whether these PU.1-bound regions with distinct *cis*-elements could be co-bound or tethered differentially by other factors in addition to PML/RARα. For this analysis we took advantage of the published Encyclopedia of DNA elements (ENCODE) project data that includes the genomic regions bound by 119 human transcription factors involving 72 different cell types [36,37]. We compiled three different PU.1-bound region sets, including the PU.1-specific with the long motif (Long), the PU.1-specific with the short motif (Short) and the PU.1&PR, and carried out overlapping analysis with 1328 ChIP-seq data sets downloaded from the UCSC ENCODE data center. As shown in Figure 4D and 4E, most of the ENCODE TFs showed significantly less preference to the Long set than the Short set and the PU.1&PR set (paired t-test p<1e-303 and p<1e-296, respectively). In particular, factors, such as MAX (21.3% vs. 37.7%) and MYC (17.8% vs. 34.4%) in NB4 cells, JUND in K562 cells (13.4% vs. 26.0%) and CTCF in HCPE (12.3% vs. 27.3%) and HBMEC (12.9% vs. 25.9%) cells showed the most distinct overlapping percentage between the Long and Short sets. In contrast, the Long set was significantly more covered by 9 ENCODE PU.1 data sets involving 4 cell types (GM12878, GM12891, HL60 and K562) than the Short and PU.1&PR sets (paired t-test p<1e-6 and p<0.0002, respectively; Figure 4F). The result suggested that the long-motif-containing sites kept more PU.1 binding stability among different cell types, consistent with their higher binding affinity to PU.1 revealed above, while the short-motif-containing sites could recruit more TFs to regulate the corresponding gene expression.

Furthermore, we wanted to know whether genes regulated by different PU.1 *cis*-elements or binding factors were functionally differentiated. Thus, we conducted Gene Ontology (GO) and Kyoto Encyclopedia of Genes and Genomes (KEGG) pathway analyses respectively on the three gene sets (Long, Short and PU.1&PR). As shown in Figure 4G, the most striking GO terms revealed in the gene set targeted by both PU.1 and PML/RARα (PU.1&PR) were highlighted by genes involved in oncogenesis and hematopoiesis. The former was represented by *JUNB, BCL2, MLLT10, FES, FOS* and *MCL1*, and the latter was represented by *RUNX1, CEBPA* and *STAT3*. Similarly, the most significant KEGG pathways revealed in this gene set were represented by those involved in acute myeloid leukemia, including *SPI1*, *RARA*, *KRAS* and so on (Figure 4H and Additional file 8: Figure S5). These results provide additional evidence that genes targeted by both PU.1 and PML/RARα are indispensable for the normal hematopoiesis, and are crucial for leukemogenesis (Figure 4G and 4H). Besides, we also noticed that the genes belong to the Long set specifically evolved in several biological processes and functional pathways, such as developmental processes and adherens junction, respectively. The Short gene set, however, showed little particularly functional enrichment, implying PU.1 may involve more extensive biological functions rather than some specific ones through the different dynamic combinations with other factors.

## Discussion

PU.1 is a master TF in myeloid differentiation, regulating numerous myeloid genes involved in hematopoiesis. Understanding *cis*-regulatory elements that interact with PU.1 may provide insights into regulatory networks underlying myeloid differentiation and related diseases. In this study, we identified 26,907 *in vivo* PU.1 binding sites in APL-derived NB4 cells by ChIP-seq. Through various analyses, we found the PU.1 binding sites were primarily represented by two types of *cis*-elements. One, with high sequence specificity and binding affinity, resides primarily in the promoter-distal regions, and a second, with strong evolutionary constraint, represents the primary PU.1 *cis*-elements in the promoter-proximal regions; the latter tend to be bound by PU.1 in association with other factors such as PML/RARα, MYC and MAX. Our findings suggest that sequence diversity of TF binding *cis*-elements is a critical mechanism by which cells interpret the genome, and contribute to distinct physiological and/or pathological function.

Physical interaction between distinct chromosome regions separated by hundreds of kilobases is thought to be important in the regulation of gene expression [38]. By analyzing the distribution of PU.1 binding sites in APL, we found that over 85% of the sites resided in chromatin regions away from promoters. Interestingly, after mapping the PU.1 binding sites to the known RefSeq genes, more than half of the promoter-targeted RefSeq genes (1,598/3,184) contained additional PU.1 binding site at non-promoter regions, suggesting the presence of complex networks of interconnected chromatin. Also, it is tempting to assume that there is a mode of action through the long-range regulation in addition to conventional models of the promoter regulation in PU.1-regulated gene expression. Such an assumption appears to be supported by recent findings in many cell types, in which long-range interactions are common for transcriptional regulation [39], and by the findings that interacting loci between transcription start sites (TSSs) and enhancers are strongly correlated with gene expression level [36]. Another example of long-range regulation is provided by estrogen receptor alpha (ERα) induced looping [40], in which the vast majority of ERα binding sites reside in non-promoter regions [41]. It has been reported that PU.1 mediated looping between promoter pIII and the distal element termed hypersensitive site 1 (HSS1) of transcriptional co-activator CIITA in B cell of mouse model [42], but whether looping is common or infrequent in the regulatory networks of PU.1 remains to be elucidated. Consistent with previous investigations at the single gene level, our study added additional 1,598 PU.1 target genes potentially regulated across long distances.

The availability of genome-wide TF binding sites benefits our investigation regarding the regulatory mechanisms underlying protein-DNA interaction and improves the accuracy for analyzing *cis*-regulatory elements. In this study, we found potential *cis*-elements of PU.1 target genes can be classified into the canonical short consensus (AG(A/G)GGAAG) and the extended long consensus ((A/G)AAAG(A/G)GGAAGTG). The shorter sequence appears to be highly conserved across species but shows less binding affinity to PU.1, whereas the extended one demonstrates high binding affinity but low evolutionary conservation. This observation is also supported by a microarray-based binding affinity study, in which the vast majority of the 104 DNA-binding proteins tested showed different binding affinities to different DNA consensuses [43]. Interestingly, potential *cis*-elements in the promoter-proximal regions of PU.1 regulated genes are predominantly represented by the short motif elements (63.4%) whereas these regions are relatively depleted of the long motif elements (14.5%). In contrast, the long motif elements primarily reside in the promoter-distal regions (including enhancers). Although it remains to be elucidated why these long and evolutionally diverse elements are predominantly located in non-promoter regions, it might be of interests to speculate that these elements may play species-specific roles in precise regulation of gene expression required for myeloid.

Of note, PML/RARα targeted PU.1 binding regions are highly enriched with the canonical short consensus (69.2%) but depleted of the extended long consensus sequence (11.6%). An intensive overlapping analysis revealed that most of the over 100 tested transcription factors showed more preference to the short-motif-containing binding regions of PU.1. Although much remains to be elucidated, it is tempting to assume that binding of PU.1 to the low affinity canonical consensus may represent an important mechanism that controls physiological or pathological process through the differential dynamic combinations with other factors, such as MYC and MAX. The extended long consensus elements are mostly distributed in non-promoter regions, and represent high affinity binding sites of PU.1. We speculate that these sites cooperate with the binding sites in promoter regions and regulate hematopoietic specific genes through long-range regulation. In the presence of the pathological protein PML/RARα, these sites are devoid of targeting by PML/RARα to a large extent, probably needed to keep certain expression levels of the genes. Indeed, 80% down-regulation of PU.1 in hematopoietic cells causes the blockage of cell differentiation [44] and restoration of PU.1 expression induces neutrophil maturation [3]. Obviously, much remains to be investigated about regulatory networks of PU.1 in myeloid differentiation or leukemogenesis. However, findings and a resource of thousands of potential *cis*-elements of PU.1 from this setting may facilitate such investigation in a more effective and efficient manner.

## Conclusions

We here describe a genome-wide characterization of *in vivo* binding sites of PU.1 in APL-derived NB4 cells. Our results demonstrate that PU.1 can regulate target genes by binding to both the promoter-proximal and distal *cis*-elements. Moreover, we reveal that structurally differentiated *cis*-elements that interact with PU.1 are functionally distinguishable in acute promyelocytic leukemia, suggesting that sequence diversity of *cis*-elements that interact with *trans*-acting factors might be a critical mechanism by which cells interpret the genome, and contribute to distinct physiological and/or pathological function.

## Materials and methods

### Cell culture

NB4 cells were cultured in RPMI 1640 medium supplemented with 10% fetal bovine serum (FBS). HEK 293T cells were cultured in Dulbecco’s modified Eagle’s medium (DMEM) supplemented with 10% FBS, in a humidified atmosphere with 5% CO_2_ at 37°C.

### Plasmid construction, cell transfection and luciferase reporter assays

Promoter and enhancer regions harboring PU.1 motifs were cloned into the pGL3-basic and pGL3-promoter vector (Promega, Madison, WI), respectively. The primers used for the plasmids constructs are listed in Additional file 9: Table S4. The *renilla* luciferase plasmid pRL-SV40 (Promega, Madison, WI) was used as control for transfection efficiency. The expression plasmid was pCMV4-PU.1. HEK 293T cells were transiently transfected using Lipofectamine 2000 (Invitrogen, Carlsbad, CA). Transfected cells were cultured for 48 hours and then assayed for luciferase activity using Dual-Luciferase Reporter Assay System reagents (Promega, Madison, WI).

### ChIP-qPCR

ChIP-qPCR was performed using Power SYBR® Green PCR Master Mix (Applied Biosystems, Foster City, CA) and ABI Prism 7900HT detection system (Applied Biosystems, Foster City, CA). The fold enrichment of the tested binding regions over the input DNA was estimated as previously described [45]. The primers used for ChIP-qPCR are listed in Additional file 10: Table S5.

### ChIP-seq and data analysis

ChIP was performed using specific antibodies according to the Affymetrix protocol as described previously [40]. ChIPed and Input DNA were sequenced with Illumina Genome Analyzer II. The 35 bp reads (or tags) were aligned (mapped) to the unmasked human reference genome (NCBI v36, hg18) using the Eland application (Illumina) allowing two mismatches. Only uniquely mapped reads were retained to further analyses. Next, MACS [16] algorithm was used to identify PU.1 binding regions. For the visualization of the enrichment level of transcription factor’s binding sites, we calculated the tag density of each ChIP-seq sample with 500 bp window, aligned it to the same coordinate and visualized them in a bar plot using the IGB (Affymetrix) program.

### Peak mapping and annotation

We used the RefSeq Genes’ database from UCSC to map and annotate the peak regions. For each peak region, we first searched the nearest RefSeq gene in both directions unless no gene was found within 50 kb. For a peak region lying within a gene, we classified it to the proximal promoter (-2 kb upstream to 1kb downstream to TSS), gene body (1 kb downstream of the TSS to the transcription end site (TES)), upstream enhancer (between at most -50 kb and -2 kb upstream to the TSS), or downstream enhancer (from TES to at most 50 kb downstream). Otherwise, we marked these peaks as distal intergenic region (>50 kb from a RefSeq gene). To avoid multiple genomic region type annotation of one peak, we uniquely mapped the peaks to genomic region type following the priority rule: proximal promoter > gene body > upstream enhancer > downstream enhancer > distal intergenic region.

### Sequence evolutionary conservation analysis

The enriched peaks were first uniquely mapped to certain genomic regions according to the above peak mapping and annotation criteria. Then, the regions were aligned at their summits from 5^′^ to 3^′^ in accordance with the orientation of the corresponding genes (if a peak belongs to the distal intergenic region, we arbitrarily assume that the peak is on the positive strand) and uniformly expanded to 3,000bp in each direction, and phastCons scores were retrieved from UCSC genome browser (http://genome.ucsc.edu) and averaged at each position.

### Statistical analysis of the known motifs with MotifScan

A position-specific frequency matrix (PSFM) similarity based motif analysis algorithm, named MotifScan, was used to analyze the enrichment of the known motifs statistically on the ChIP-seq data. Fold enrichment and Z-score were used to assess the significance of motif enrichment. MotifScan was performed as follow:

1) The acquisition of motif PSFM

The motif PSFM can be from the result of *de novo* motif analysis on a set of DNA sequence data. In this study, the long motif PSFM of PU.1 was from the result of AMD [34], an automated motif discovery tool using stepwise refinement of gapped consensuses, analysis on the highly enriched PU.1 occupancy sites of ChIP-seq data set. It also could be from some motif information database directly and the TRANSFAC database was used in this study.

2) The calculation of similarity between a motif and a DNA sequence

Given a motif with length *l* bp, the similarity (S) for a DNA sequence with equal length was calculated as follow:

$$S=\frac{\sum_{j=1}^{l}I_{j}\left(2\sqrt{P_{\mathrm{ij}}}-1\right)}{\sum_{j=1}^{l}I_{j}\left(2\sqrt{P_{j}^{\max}}-1\right)}$$

Where *I*_*j*_ indicates the information content of the motif *j*-th column, *P*_*ij*_ is the frequency of a particular letter *i* in the *j*-th column and $P_{j}^{\max}$ is the frequency of a specific letter with the maximum value in the *j*-th column. Details described as follow:

$$I_{j}=\left(\sum_{i\in \left\{A,T,G,C\right\}}P_{\mathrm{ij}}\log\frac{P_{\mathrm{ij}}}{Q_{i}}\right)$$

Where:

••*P*_*ij*_ – The frequency of a particular letter *i* in the *j*-th column (i. e. if G occurred 3 out of 6 times in an alignment column, this would be 0.5). We define *P*_*ij*_ log *P*_*ij*_ = 0, when *P*_*ij*_ = 0.

••*Q*_*i*_ – The expected frequency of a letter *i*. This is an optional argument, usage of which is left at the user’s discretion. By default, it is automatically assigned to 0.25 = 1/4 for a nucleic acid alphabet. This is for getting the information content without any assumption of prior distributions.

3) The computation of fold enrichment (F) and Z-score (Z)

For a specific motif, the sequence with a similarity higher than the threshold was marked as a matched sequence. Suppose that the total number of matched sequences was *X* in a data set with total effective sequence length *L* bp (repeat masked). Then the random variable *X* followed the binomial distribution with parameters n and p, written as *X* ∼ *B*(*n*, *p*), where *n* ≈ *L*, and *p* was the possibility that each bp contained a matched sequence. In this case, n was very large generally. Meanwhile, n*p and n*(1-p) were large enough. Thus, to simplify the calculation, we used the normal distribution *X* ∼ *N*(*np*, *np*(1 − *p*)) to excellently approximate the binomial distribution. Subsequently, we further simplified the 1–*p* as 1, since the *p* was small generally. Therefore, the expected value (E) and the variance (*σ*^2^) of *X* would be the same.

Next, the total number of matched sequences was counted in a background data set and a sample data set, respectively. Suppose that the total number of matched sequences is *N*_*s*_ in a sample data set with total effective sequence length *L*_*s*_ (bp), and *N*_*c*_ in a background data set with total effective sequence *L*_*c*_ (bp). We calculated the expected number of matched sequences in the sample data set as $E=\frac{N_{c}\times L_{s}}{L_{c}}$ Followed the above simplified formula, the fold enrichment and Z-score were calculated as F=NsE and $Z=\frac{N_{s}-E}{\sqrt{E}}$, respectively.

### Percentage calculation of the motif-containing peaks

For a specific motif, if a peak region contained at least one matched sequence, then we marked this peak as a motif-containing one. The percentage of certain motif-containing peaks was calculated as the number of motif-containing ones divided by all the number of peaks. The similarity was calculated as the same as in MotifScan algorithm. In this study, to enhance the accuracy and specificity, the similarity threshold for the “long” PU.1 motif was set at 0.8 and that for the “short” PU.1 motif and RARE half was set at 0.9. We first excluded all the long PU.1 motif-containing peaks when calculating the only short PU.1 motif-containing ones, as the short PU.1 motif was nearly covered by the long one.

## Additional files

## Abbreviations

AML: Acute myeloid leukemia; APL: Acute promyelocytic leukemia; ChIP: Chromatin immunoprecipitation; ChIP-seq: Chromatin immunoprecipitation coupled with deep sequencing; TFs: *trans*-acting factors; PML/RARα: Promyelocytic leukemia-retinoic acid receptor alpha; ATRA: all *trans* retinoid acid; FDR: False discovery rate; RefSeq: Reference sequences; RNAPII: RNA polymerase II; ER: Estrogen receptor; RARE: Retinoic acid response element; RAREh: RARE half; UCSC: University of California Santa Cruz; ENCODE: Encyclopedia of DNA Elements; GO: Gene ontology; KEGG: Kyoto encyclopedia of genes and genomes; HSS1: Hypersensitive site 1; PSFM: Position-specific frequency matrix; TSS: Transcription start site; TES: Transcription end site

## Competing interests

The authors declare that they have no competing interests.

## Authors’ contributions

MXQ designed the study, performed experiments, conducted the analyses and wrote the manuscript; WJ, XHZ, XHJ, and XWY performed experiments; YZD participated in the design of the study and reviewed the manuscript; KKW and JZ designed the study, interpreted the results and wrote the manuscript. All authors read and approved the final manuscript.

## Supplementary Material

Additional file 1: Figure S1 Identification of PU.1 binding sites based on different FDR levels. Click here for file

Additional file 2: Table S1 Significantly enriched PU.1 ChIP regions in NB4 cells. Click here for file

Additional file 3: Figure S2 Representative PU.1 target genes identified through ChIP-seq analysis. Click here for file

Additional file 4: Table S2 Chromosomal distribution of PU.1 binding sites. Click here for file

Additional file 5: Figure S3 Representative myeloid differentiation-required genes targeted by PU.1. Click here for file

Additional file 6: Figure S4 PU.1 motif identified by *de novo* motif discovery method MEME. Click here for file

Additional file 7: Table S3 Significantly enriched PML/RARα ChIP regions in NB4 cells. Click here for file

Additional file 8: Figure S5 KEGG pathway acute myeloid leukemia. Click here for file

Additional file 9: Table S4 The primers used for the plasmids constructs. Click here for file

Additional file 10: Table S5 The primers used for ChIP-qPCR. Click here for file
