# Supplementary material for: Structurally differentiated cis-elements that interact with PU.1 are functionally distinguishable in acute promyelocytic leukemia
Source: J Hematol Oncol. 2013 Apr 2;6:25. doi: 10.1186/1756-8722-6-25 (PMC3618267; doi:10.1186/1756-8722-6-25)

**Figure S1. Identification of PU.1 binding sites based on different FDR levels**

FDR 0.1% was chose to determine the final PU.1 binding sites according to the slope transformation.


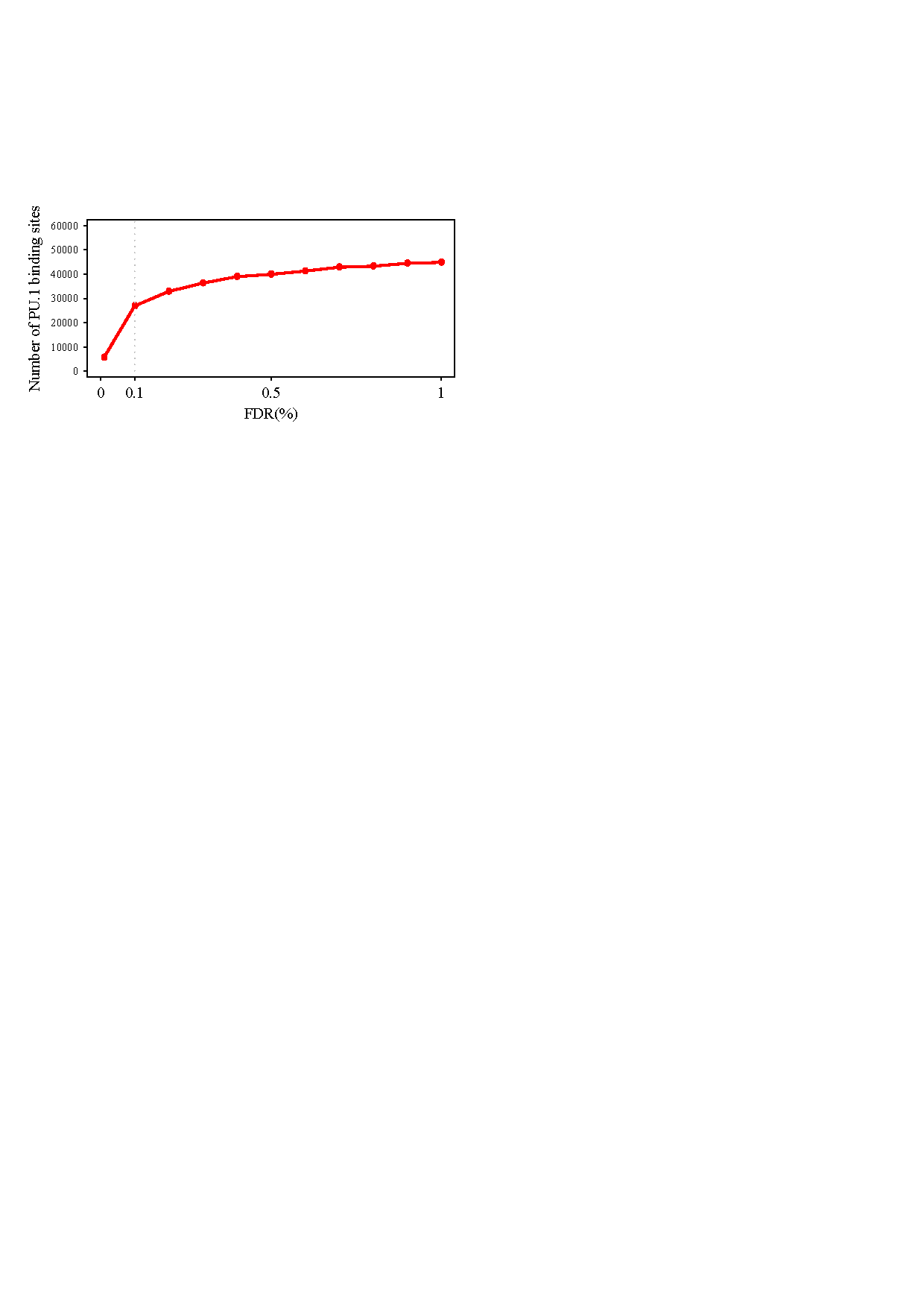

Supplement: Additional file 1: Figure S1 — Identification of PU.1 binding sites based on different FDR levels. [file 1756-8722-6-25-S1.doc]
