# Supplementary material for: Structurally differentiated cis-elements that interact with PU.1 are functionally distinguishable in acute promyelocytic leukemia
Source: J Hematol Oncol. 2013 Apr 2;6:25. doi: 10.1186/1756-8722-6-25 (PMC3618267; doi:10.1186/1756-8722-6-25)
Supplement: Additional file 4: Table S2 — Chromosomal distribution of PU.1 binding sites. [file 1756-8722-6-25-S4.doc]

**Table S2. Chromosomal** distribution of PU.1 binding sites

| **Chromosome** | **Genes** | **Length (bp)** | **PU.1** | **STAT1** | **ER** | **FOXA1** | **RNAPII** | **CTCF** | **GATA1** | **GATA2** |
| --- | --- | --- | --- | --- | --- | --- | --- | --- | --- | --- |
| chr1 | 2,110 | 247,249,719 | 2,535 | 4,591 | 398 | 1,338 | 1,059 | 1,405 | 822 | 3,033 |
| chr2 | 1,297 | 242,951,149 | 2,197 | 2,984 | 295 | 1,419 | 898 | 986 | 361 | 1,428 |
| chr3 | 1,061 | 199,501,827 | 1,638 | 2,965 | 256 | 1,035 | 674 | 662 | 321 | 1,270 |
| chr4 | 765 | 191,273,063 | 1,204 | 1,360 | 111 | 380 | 539 | 435 | 203 | 742 |
| chr5 | 863 | 180,857,866 | 1,446 | 2,790 | 247 | 646 | 588 | 663 | 275 | 1,041 |
| chr6 | 1,092 | 170,899,992 | 1,519 | 2,601 | 262 | 1,014 | 593 | 688 | 467 | 1,822 |
| chr7 | 939 | 158,821,424 | 1,335 | 1,945 | 255 | 530 | 700 | 679 | 372 | 1,442 |
| chr8 | 690 | 146,274,826 | 1,048 | 2,059 | 244 | 807 | 502 | 575 | 234 | 818 |
| chr9 | 844 | 140,273,252 | 1,224 | 2,051 | 170 | 578 | 610 | 574 | 205 | 710 |
| chr10 | 762 | 135,374,737 | 1,221 | 1,981 | 213 | 729 | 449 | 689 | 277 | 948 |
| chr11 | 1,258 | 134,452,384 | 1,312 | 1,808 | 142 | 502 | 626 | 905 | 299 | 1,087 |
| chr12 | 1,028 | 132,349,534 | 1,526 | 2,280 | 163 | 668 | 663 | 718 | 237 | 977 |
| chr13 | 335 | 114,142,980 | 951 | 850 | 59 | 340 | 172 | 272 | 50 | 211 |
| chr14 | 633 | 106,368,585 | 802 | 1,062 | 112 | 566 | 373 | 440 | 102 | 409 |
| chr15 | 603 | 100,338,915 | 1,024 | 1,435 | 86 | 261 | 256 | 548 | 174 | 642 |
| chr16 | 844 | 88,827,254 | 1,064 | 1,572 | 102 | 264 | 592 | 531 | 232 | 757 |
| chr17 | 1,191 | 78,774,742 | 1,227 | 2,004 | 133 | 512 | 653 | 769 | 297 | 1,044 |
| chr18 | 280 | 76,117,153 | 334 | 725 | 26 | 122 | 94 | 257 | 83 | 340 |
| chr19 | 1,325 | 63,811,651 | 980 | 1,346 | 85 | 206 | 764 | 615 | 293 | 940 |
| chr20 | 578 | 62,435,964 | 747 | 1,124 | 150 | 269 | 344 | 438 | 152 | 538 |
| chr21 | 236 | 46,944,323 | 302 | 541 | 66 | 230 | 127 | 130 | 81 | 293 |
| chr22 | 502 | 49,691,432 | 602 | 514 | 36 | 125 | 178 | 420 | 84 | 257 |
| chrX | 836 | 154,913,754 | 669 | 911 | 54 | 347 | 248 | 321 | 105 | 404 |

The first column lists each chromosome symbol. The second column lists the length of each chromosome in base pairs. The third column lists the number of genes found in each chromosome. The fourth to eleventh columns list the number of binding sites of PU.1, STAT1, ER, FOXA1, RNAPII, CTCF, GATA1 and GATA2 found in each chromosome, respectively.
