# Supplementary material for: Structurally differentiated cis-elements that interact with PU.1 are functionally distinguishable in acute promyelocytic leukemia
Source: J Hematol Oncol. 2013 Apr 2;6:25. doi: 10.1186/1756-8722-6-25 (PMC3618267; doi:10.1186/1756-8722-6-25)
Supplement: Additional file 5: Figure S3 — Representative myeloid differentiation-required genes targeted by PU.1. [file 1756-8722-6-25-S5.doc]

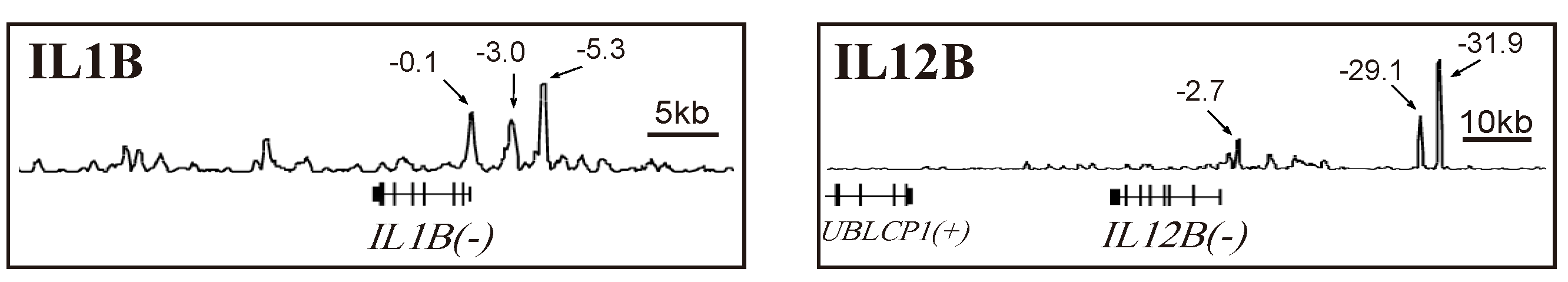


**Figure S3.** **Representative myeloid differentiation-required genes targeted by PU.1**

(Arrows) ChIP-seq peak locations relative to the start site of the respective PU.1 target gene (kb).
