# Supplementary material for: Structurally differentiated cis-elements that interact with PU.1 are functionally distinguishable in acute promyelocytic leukemia
Source: J Hematol Oncol. 2013 Apr 2;6:25. doi: 10.1186/1756-8722-6-25 (PMC3618267; doi:10.1186/1756-8722-6-25)
Supplement: Additional file 6: Figure S4 — PU.1 motif identified by de novo motif discovery method MEME. [file 1756-8722-6-25-S6.doc]

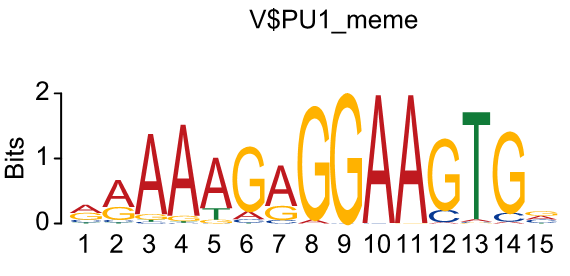


**Figure S4.** **PU.1 motif identified by *de novo* motif discovery method MEME**

Sequence logos were showed for significant PU.1 motifs. The logo was obtained by de novo motif finding using MEME.
