# Supplementary material for: Structurally differentiated cis-elements that interact with PU.1 are functionally distinguishable in acute promyelocytic leukemia
Source: J Hematol Oncol. 2013 Apr 2;6:25. doi: 10.1186/1756-8722-6-25 (PMC3618267; doi:10.1186/1756-8722-6-25)
Supplement: Additional file 8: Figure S5 — KEGG pathway acute myeloid leukemia. [file 1756-8722-6-25-S8.doc]

**
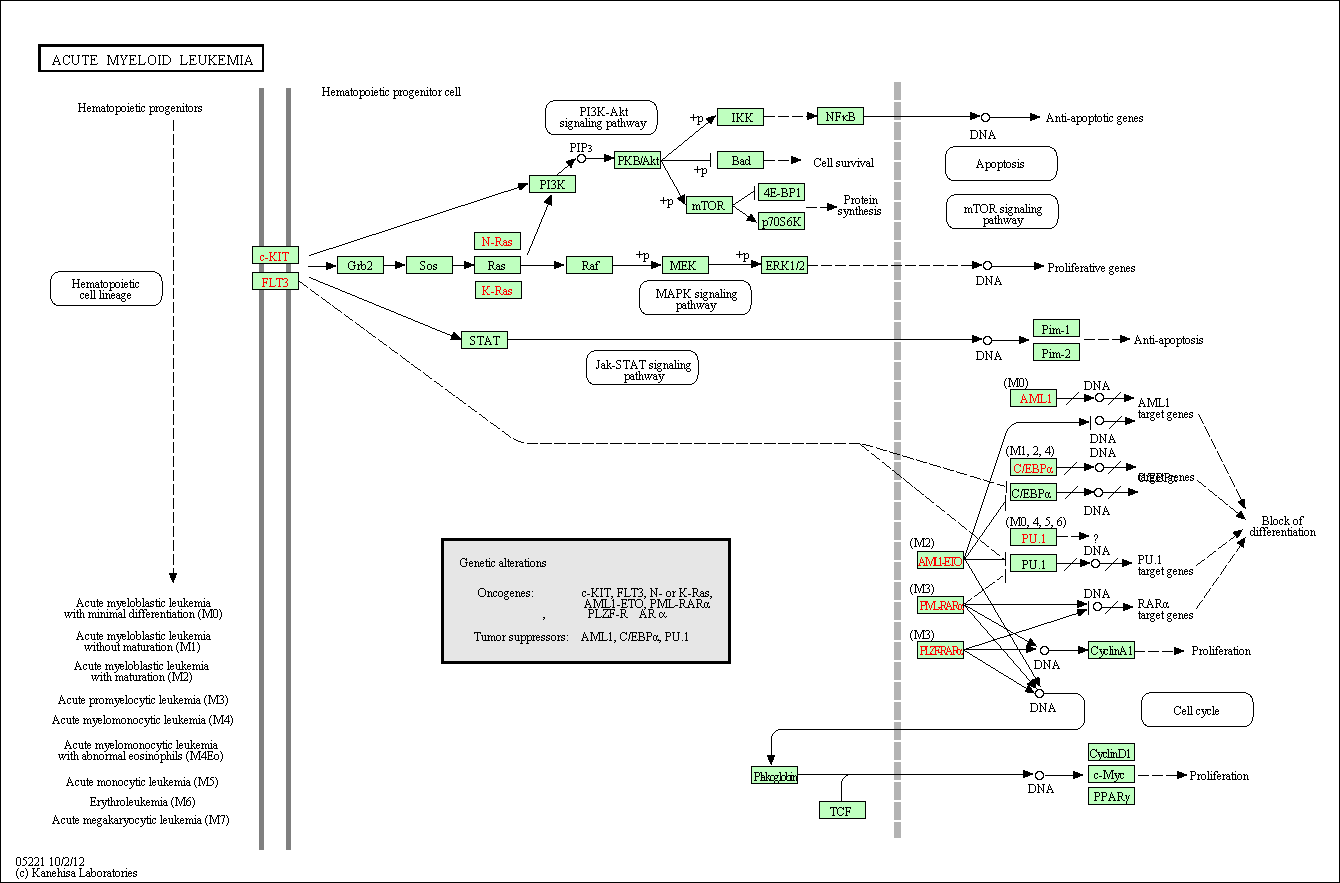
**

**Figure S5. KEGG pathway acute myeloid leukemia**

### Pathway information was generated by Kyoto Encyclopedia of Genes and Genomes(KEGG). The genes overlapping with the PU.1&PR gene set were shown in red.
