# Supplementary material for: Structurally differentiated cis-elements that interact with PU.1 are functionally distinguishable in acute promyelocytic leukemia
Source: J Hematol Oncol. 2013 Apr 2;6:25. doi: 10.1186/1756-8722-6-25 (PMC3618267; doi:10.1186/1756-8722-6-25)
Supplement: Additional file 9: Table S4 — The primers used for the plasmids constructs. [file 1756-8722-6-25-S9.doc]

**Table S4. The primers used for the plasmids constructs**

| **Symbol of gene nearby** | **Chromosome** | **F-primer (5’-3’)** | **R-primer (5’-3’)** |
| --- | --- | --- | --- |
| *NCF4* | chr22 | ATAAAGGTGGGGCTTGATGGGC | AGTCCCCAGGAGCTGCCTTTG |
| *NCF2* | chr1 | CCACCAGGGACATGATTAGGTA | AGGGACAGAAACAATGTCACAG |
| *IL1B* | chr2 | GCTCATCTGGCATTGATCT | GACTCCCTTAGCACCTAGTTG |
| *BTK* | chrX | TCAACTGTGTGTCCCTGAGAC | GAGGCAGAGGCTGCTTTCT |
| *PTPRC* | chr1 | TCCCTCATCAGTAGCGCCAA | AACAGCATGCGTCCTTCTGAA |
| *NTS* | chr12 | TGGAACCACTAAACATTTGC | CCTAAGAGAATGAATGAGGATG |
| *RGS18* | chr1 | ACATTTTAACACACAGTCCACAC | GGCTTCTTTGCTTGTTTCTTC |
| *CD163* | chr12 | GAAATGACAAATGCTGTGTCTC | CTTCTCGTTACTCTAACCCAACA |
