# Supplementary material for: Structurally differentiated cis-elements that interact with PU.1 are functionally distinguishable in acute promyelocytic leukemia
Source: J Hematol Oncol. 2013 Apr 2;6:25. doi: 10.1186/1756-8722-6-25 (PMC3618267; doi:10.1186/1756-8722-6-25)
Supplement: Additional file 10: Table S5 — The primers used for ChIP-qPCR. [file 1756-8722-6-25-S10.doc]

**Table S5. The primers used for ChIP-qPCR**

| **Symbol of gene nearby** | **Chromosome** | **F-primer (5’-3’)** | **R-primer (5’-3’)** |
| --- | --- | --- | --- |
| *CSF3R* | chr1 | GCTCGGAAGGTGTTGCAATC | CAGCTCTCCATCAAGCCCAA |
| *JUNB* | chr19 | TAATGCCTCCTTCCCGCTTA | GACACCTGACCCAGGAATTGA |
| *CCL3* | chr17 | CATCAACCCTATCCCTGTGGT | CAACCCAAGGCTATTCTTAGTCAG |
| *PPBP* | chr4 | TTCAGGTGCTCCTAAAGTTTCTATT | CCGTTCAGGAAGGTAAGAACTAGAC |
| *HHEX* | chr10 | GGTTCAACAGGCTTGTGCAGT | CCGGCTATCAGAAGTCGAGTG |
| *LMO2* | chr11 | GCACTTATAACTGTTCAGACC | CCAATGCTATGTAACACACAC |
| *NCF2(E)* | chr1 | TCTGTGCGACTCGTGCTAGTTC | GGTGCTAAGACTTGCATGCACA |
| *PLA2G4A* | chr1 | GCACTCATGGAATTTAGGACTGA | AGAACGCTCTCTTCTCATAGAAGC |
| *UBL3(E)* | chr13 | TGCTGAGCGATTTGGAGAAGT | TGTCCCCTCAAGCTACCATGAT |
| BAT5 | chr6 | CAGCTCCGGAAGTCAAAGTTGT | TCCCCTGATTTTCTGGTCCTG |
| *BLNK(E)* | chr10 | GGCCCTGACTGATGGAAATTAC | CAGCAGGTGACCATCCCTTTAG |
| *ITGA6* | chr2 | GGCTGAGTACACTCTGCAGTGAG | CGTGAACATCATCAAGGGTCC |
| WBSCR28 | chr7 | CACCTTCTAATGTTCCCTTCTCAG | AGGAACCCCACTTTTACTGTAGAG |
